# Endogenous H_2_S producing enzymes are involved in apoptosis induction in clear cell renal cell carcinoma

**DOI:** 10.1186/s12885-018-4508-1

**Published:** 2018-05-24

**Authors:** Jan Breza, Andrea Soltysova, Sona Hudecova, Adela Penesova, Ivan Szadvari, Petr Babula, Barbora Chovancova, Lubomira Lencesova, Ondrej Pos, Jan Breza, Karol Ondrias, Olga Krizanova

**Affiliations:** 1 Department of Urology with Kidney Transplant Center, University Hospital, Faculty of Medicine, Bratislava, Slovakia; 2 Institute of Clinical and Translational Research, Biomedical Research Center, SAS, Bratislava, Slovakia; 3 Department of Molecular Biology, Faculty of Natural Sciences, Comenius University, Bratislava, Slovakia; 4 Department of Physiology, Faculty of Medicine, Masaryk University, Brno, Czech Republic

**Keywords:** Cystathionine-β-synthase, Cystathionine γ-lyase, 3-Mercaptopyruvate sulfurtransferase, Apoptosis, Clear cell renal cell carcinoma

## Abstract

**Background:**

Knowledge about the expression and thus a role of enzymes that produce endogenous H_2_S - cystathionine-β-synthase, cystathionine γ-lyase and mercaptopyruvate sulfurtransferase - in renal tumors is still controversial. In this study we aimed to determine the expression of these enzymes relatively to the expression in unaffected part of kidney from the same patient and to found relation of these changes to apoptosis. To evaluate patient’s samples, microarray and immunohistochemistry was used.

**Methods:**

To determine the physiological importance, we used RCC4 stable cell line derived from clear cell renal cell carcinoma, where apoptosis induction by a mixture of five chemotherapeutics with/without silencing of H_2_S-producing enzymes was detected. Immunofluorescence was used to determine each enzyme in the cells.

**Results:**

In clear cell renal cell carcinomas, expression of H_2_S-producing enzymes was mostly decreased compared to a part of kidney that was distal from the tumor. To evaluate a potential role of H_2_S-producing enzymes in the apoptosis induction, we used RCC4 stable cell line. We have found that silencing of cystathionine-β-synthase and cystathionine γ-lyase prevented induction of apoptosis. Immunofluorescence staining clearly showed that these enzymes were upregulated during apoptosis in RCC4 cells.

**Conclusion:**

Based on these results we concluded that in clear cell renal cell carcinoma, reduced expression of the H_2_S-producing enzymes, mainly cystathionine γ-lyase, might contribute to a resistance to the induction of apoptosis. Increased production of the endogenous H_2_S, or donation from the external sources might be of a therapeutic importance in these tumors.

## Background

Hydrogen sulfide (H_2_S), a third gasotransmitter, is involved in many physiological and pathophysiological processes. Recent studies indicate that H_2_S might have both pro-cancer and anti-cancer effects. Nevertheless, some controversy exists on the role of H_2_S in cancer development and progression, probably due to differences in solid tumors. It was already reported that variety of H_2_S-releasing compounds could inhibit growth and metastasis of different tumors, e.g. sulfide salts, diallyl trisulfide, sulforaphane, etc. [1–3]. In mammals, H_2_S can be also produced endogenously from the metabolism of L-cysteine and homocysteine by the catalysis of three enzymes, termed cystathionine γ-lyase (CSE), cystathionine β-synthase (CBS) and 3-mercaptopyruvate sulfurtransferase (MPST). While CSE and CBS are cytosolic enzymes, MPST is localized in mitochondria.

All three enzymes are expressed in different cancer cells, although their expression differs markedly according to a type of cancer (for review see [4]). CBS was shown to be up-regulated in colon cancer cells [5], ovarian cancer cells [6], or breast adenocarcinoma [7]. The expression level of CBS mRNA is low in hepatocellular carcinoma [8] or gastric and colorectal cancers [9]. Varying expression levels of CBS/CSE were found in human prostate stromal and epithelial compartments [10]. In another study, reduced expression of H_2_S-producing enzymes was observed in human prostate cancer tissues and also in prostate cancer cells [11]. Expression of the CSE, but not CBS was significantly reduced in prostate cancer tissue versus tissue from normal prostate [12]. On the other hand, Sekiguchi et al. [13] reported that endogenous H_2_S produced by CSE may contribute to the proliferation of gastric cancer AGS cells, most probably through anti-apoptotic actions.

Significance of the MPST that is localized in mitochondria has not been well-ackowledged up to now. MPST was expressed neither in human prostate adenocarcinoma, nor in normal prostate tissues [12]. In melanoma, MPST expression was always extremely variable in the human specimens analyzed [from nevi to metastasis; [14]]. In kidney, MPST has been detected, although the significance of this enzyme in kidney physiology and pathophysiology is not completely elucidated yet [15]. Interestingly, MPST functions more efficiently at high pH [11].

Renal cell carcinoma belongs to the common urologic tumors. These tumors are formed from heterogeneous epithelium of renal tubules. RCC is not a single entity, but rather comprises a population of tumors that originate from the highly heterogeneous epithelium of renal tubules. Histological subtypes of RCC include clear cell renal cell carcinoma (ccRCC), chromophobe collecting duct carcinoma, papillary carcinoma, and other unclassified carcinomas [16]. The ccRCC frequently carries von Hippel-Lindau tumor-suppressor gene mutations or loss, which causes uncontrolled hypoxia-inducible factor activation. Variety of chemotherapeutics is used to treat cancer, nevertheless, sometimes the effort is impeded due to drug resistance. Susceptibility of the chemotherapy might decrease over the time due to distinct mechanisms, such as DNA mutations, or metabolic changes that can promote drug inhibition and degradation.

From all above-mentioned results it is apparent that endogenous H_2_S production depends on the type of cancer. A number of studies have evidenced the role of H_2_S in inducing cell death and exerting both pro- and anti-apoptotic activity in cultured cells [17, 18]. Recently, Shackelford et al. [19] found that CBS protein levels were increased in ccRCCs in comparison to benign renal cortex and suggested that CBS and H_2_S likely play a role in malignant and benign neoplastic renal disease. However, an expression of other H_2_S producing enzymes, which are present in kidney [15], CSE and MPST, is unknown in ccRCC. Therefore, the main goal of this work was to evaluate expression of CBS, CSE and MPST in patients diagnosed with renal cancer and try to associate results with apoptosis induction in vitro. Particularly, we determined the expression and localization of CSE, CBS and MPST in renal carcinomas of 26 patients compared to matching unaffected part of kidney. Also, we investigated the possible participation of these enzymes on the apoptosis induction in RCC4 stable cell line derived from ccRCC.

## Methods

### Patients

Twenty six patients (16 male/10 female, average age 57.1 ± 14.8 yrs) were admitted for surgery. All patients had histopathologically confirmed renal carcinoma and underwent radical nephrectomy. Two tumors were detected as angiomyolipoma, three tumors as papillary carcinoma and rest of tumors was ccRCC. After nephrectomy, tumor mass and also corresponding healthy part of tissue was immediately taken into the RNA Latter® and kept at 4 °C until isolation. Tumor sample (cca 0.5cm^2^) was cut off from the outer part of tumor and corresponding unaffected tissue was taken from the distinct part of extirpated kidney. For immunohistochemistry and TUNNEL assay, paraffin blocks were used. TUNEL assay was performed using In Situ Cell Death Detection Kit, Fluorescein (Roche, Sigma-Aldrich, USA) according to manufacturer’s instructions. Tumor nuclear grading was based on the criteria of Fuhrman by a single pathologist. The RCC Fuhrman grades were grade I in 2 samples, grade II in 11 samples, grade III in 2 sample and grade IV 3 samples, tumor grade of the rest of patients was unknown.

All subjects gave informed written consent and the study was approved by the Ethics Committee of the Biomedical Research Center SAS nr. EK/BmV-01/2016 and University Hospital Bratislava, Slovakia, nr. EK 131/17, in agreement with the Ethical guidelines of the Declaration of Helsinki as revised in 2000.

### Microarray assays

500 ng of total RNA was transcribed into cDNA. Subsequently labeling reaction was performed using Cy3-dCTP (unaffected tissue samples) and Cy5-dCTP (tumor samples) to obtained cRNA. Further, microarray was performed as described in Soltysova et al. [20].

### Cell culture and treatments

In order to determine the effect of CBS, CSE and MPST in ccRCC tumors, RCC4 stable cell line (Sigma Aldrich, USA; passages 5–10) derived from clear cell renal cell carcinoma was used. Cells were grown in Dulbeco’s minimal essential medium (Sigma Aldrich, USA), supplemented with 10% fetal bovine serum and penicilin/streptomycin mixture. For experiments, cells were plated on 6-well plates or coverslips coated with polylysine and group of cells was treated with apoptosis inducer kit (AIK; Calbiochem, Merck, Darmstadt, Germany) diluted to 1:1000 as recommended by the producer. AIK is composed from the following inducers – Actinomycin D, Camptothecin, Cycloheximide, Dexamethasone, and Etoposide. Cells were treated also with D,L- propargyl glycine (PGG; 1 mM [21, 22]; Cayman Chemicals), O-(Carboxymethyl) hydroxylamine hemihydrochloride (AOAA; 10 μM [23]; Sigma Aldrich), or H_2_S producing enzymes were silenced by the procedure described in Hudecova et al. [2] using SMARTpool ONTARGETplus CBS siRNA (Dharmacon, L-008617-00-0005), SMARTpool ON-TARGETplus Cth siRNA (Dharmacon, L-064123-01-0005), SMARTpool ONTARGETplus MPST siRNA (Dharmacon, L-010119-00-0005). As a negative control (scrambled), ON-TARGETplus NON-targeting Pool siRNAs were used (Dharmacon, USA).

### Immunofluorescence

RCC4 cells grown on glass coverslips were fixed in ice-cold methanol. Immunofluorescence was performed as described in Hudecova et al. [2] with following primary antibodies: anti-CBS rabbit polyclonal antibody (ABGENT, USA), anti-CSE rabbit polyclonal antibody (antibodies-online), and anti-MPST rabbit polyclonal antibody (Abcam, UK) diluted 1:50 and 1:100 in PBS with 1% BSA.

### Detection of apoptosis with Annexin-V-FLUOS

After the AIK treatment, RCC4 cells were gently scraped and pelleted at 100 x g for 5 min. Apoptosis was performed as described in Hudecova et al. [2].

### Western blot analysis

Whole procedure was performed as it is described in detail in Hudecova et al. [2]. To detect CBS, CSE and MPST proteins, anti-CBS rabbit polyclonal antibody (ABGENT, USA), anti-CSE rabbit polyclonal antibody (antibodies-online), and anti-MPST rabbit polyclonal antibody (Abcam, UK). As a housekeeper, we used β-actin mouse monoclonal [AC-15] antibody (ab6276, Abcam, UK). An enhanced chemiluminescence detection system (LuminataTM Crescendo Western HRP Substrate, Millipore) was used to detect the bound antibodies.

## Results

**Fig. 1:**
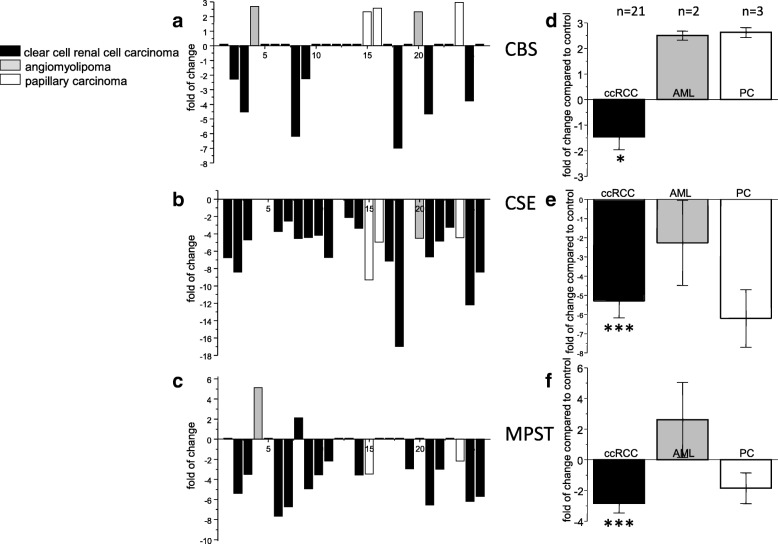
Expression status of CBS (**a**), CSE (**b**) and MPST (**c**) in renal tumors compared to non-affected kidney tissue from the same patient. From 26 renal tumors, 2 were determined as angiomyolipoma (AML; gray columns), 3 as papillary carcinoma (PC; white columns) and 21 as clear cell renal cell carcinoma (ccRCC; black columns). CBS was downregulated, or not changed in all tumors of renal cell clear cell carcinoma, while in angiomyolipoma and papillary carcinoma CBS was upregulated (**a**). CSE mRNA was downregulated in all types of tumors compared to corresponding healthy part of kidney (**b**). MPST mRNA was downregulated in almost all samples of clear cell renal cell carcinoma (**c**; except of one). Results are displayed as a fold of change to the expression of healthy tissue. In ccRCC, overall change in CBS (**d**), CSE (**e**) and MPST (**f**) from 21 patients revealed significant down-regulation, when compared to the healthy kidney tissue from the same patients. Since groups of angiomyolipoma and papillary carcinoma were negligible, we did not calculate statistical significance for these types of tumors. Results are displayed as mean ± S.E.M. and represent an average of 21patients. Statistical significance compared to corresponding controls represents * *p* < 0.05 and *** - *p* < 0.0001

**Fig. 2:**
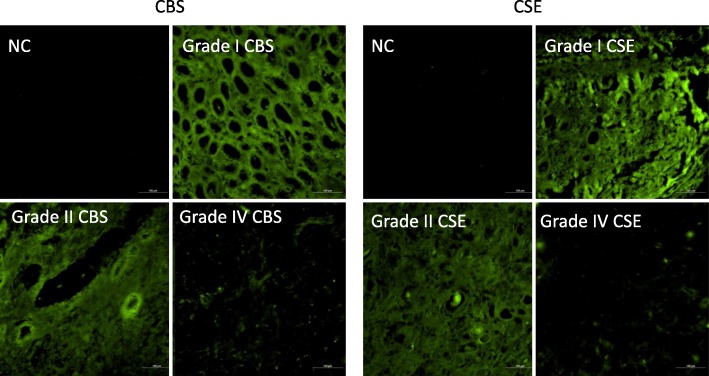
Immunohistological staining of CBS and CSE in ccRCC tumors according to a grade of tumor. Staining with primary antibody against CBS or CSE was decreased in higher grades. NC – negative control, where primary antibody was omitted. Scale bar represents 100 μm

**Fig. 3:**
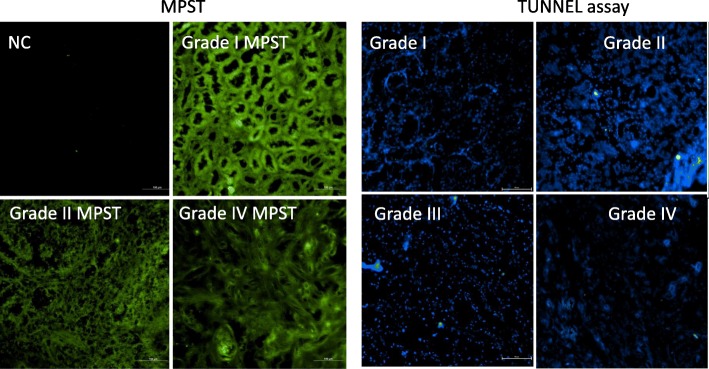
Immunohistological staining of MPST in ccRCC tumors according to a grade of tumor and determination of apoptosis by TUNNEL assay. Staining with primary antibody against MPST was slightly decreased in higher grades. In samples from all grades, apoptosis was not detectable. NC – negative control, where primary antibody was omitted. Scale bar represents 100 μm

**Fig. 4:**
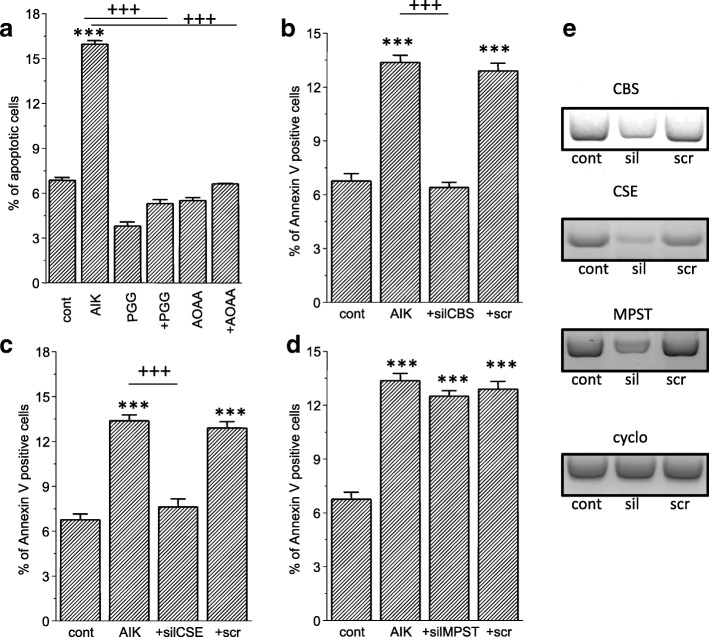
Determination of apoptosis in RCC4 cells. In a group of cells with induced apoptosis (AIK), percentage of apoptotic cells increased significantly compared to control, untreated cells (cont). Blocking with CBS and CSE blockers AOAA and PGG (**a**) revealed prevention of apoptosis induction. When CBS (**b**), CSE (**c**) or MPST (**d**) were silenced prior the apoptosis induction, apoptosis was not induced. Effectivity of silencing of CBS was ~ 60%, CSE ~ 70% and MPST ~ 50%, as determined by the gene expression. Representative gels are shown in section (**e**), where scr is scrambled siRNA. Results are displayed as mean ± S.E.M. and represent an average of 6 parallel from two independent cultivations. Statistical significance compared to control represents *** - *p* < 0.0001 and statistical significance compared to AIK treated cells represents +++ − *p* < 0.0001

**Fig. 5:**
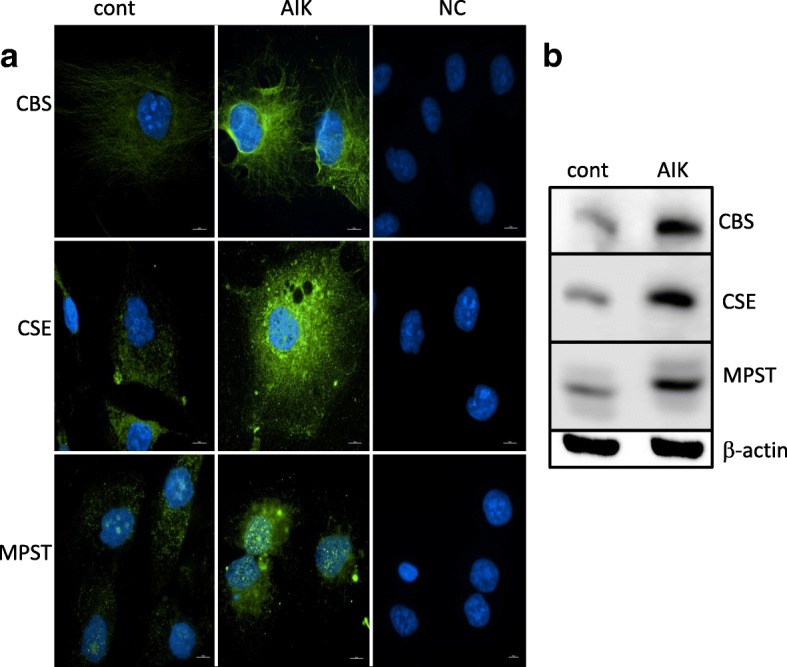
Immunofluorescence (**a**) and Western blot analysis (**b**) of CBS, CSE and MPST in control RCC4 cells (cont) and cells with induced apoptosis (AIK). In group of AIK, rapid upregulation mainly of CBS and CSE was detectable (**a**, green signal). Signal of MPST seems to be not increased so much. Nuclei were counterstained with DAPI (blue signal). Scale bar represents 100 μM. Upregulation of CBS, CSE and MPST observed by immunofluorescence was verified by Western blot analysis (**b**). All three enzymes were upregulated due to AIK treatment

Gene expression of the CBS, CSE and MPST from renal tumors was compared in 26 patients relatively to unaffected part of kidney from the same patient. We observed 2.3–2.7-fold increase in CBS expression in angiomyolipoma and 2.3–3.0-fold increase in papillary carcinoma. From 21 patients with ccRCC, CBS expression was not changed in 14 patients and was downregulated 2.3–7.0-times in 7 patients (Fig. 1a). CSE expression was downregulated in all patients with renal tumors and not changed in 3 patients with ccRCC and one patient with angiomyolipoma (Fig. 1b). Decrease in CSE expression in ccRCC was 2.1–17.0-fold and in papillary carcinoma 4.5–9.3-fold compared to corresponding non-affected tissue from the same patient. Increase in MPST expression was observed in angiomyolipoma tumor of one patient and no change in MPST was detected in another angiomyolipoma. In ccRCC, we observed increase in MPST expression in one tumor, no change in 7 tumors and 2.2–9.0-fold decrease in 15 tumors (Fig. 1c). Overall change in CBS (Fig. 1d), CSE (Fig. 1e) and MPST (Fig. 1f) revealed significant down-regulation in ccRCC. Since groups of angiomyolipoma and papillary carcinoma were negligible, we did not calculate statistical significance for these types of tumors. Although in our patients the size of tumors increased by a grade (grade I – 3.5 ± 1.47 cm, *n* = 2; grade 2–6.2 ± 0.95 cm, *n* = 9; grade IV – 9.25 ± 0.73 cm, *n* = 3), immunohistochemical staining with anti-CBS antibody suggested a rapid decrease due to an increased grade of tumor (Fig. 2). Similar result was observed using immunohistochemistry with anti-CSE antibody (Fig. 2, right). Also, MPST signal was decreased in higher grades (Fig. 3, right), although decrease in the signal intensity was not so dramatic as in CBS or CSE-stained slices. As determined by a TUNNEL assay, apoptosis was almost absent in any grade of ccRCC (Fig. 3, left). However, although these results are interesting, they should be verified by a larger amount of tumors. In order to evaluate a potential role of CBS, CSE and MPST in the apoptosis induction, apoptosis was induced in RCC4 cells, where individual endogenous enzymes were blocked by antagonists (PGG or AOAA), or silenced by appropriate siRNA (Fig. 4). Apoptosis was induced by AIK (as described in Material and Methods). When cells were treated in parallel with nonspecific blocker of CBS – AOAA, no apoptosis due to AIK treatment occurred (Fig. 4a). Also, when CBS was silenced and cells were treated with AIK, apoptosis was not induced (Fig. 4b). When CSE was blocked either with a specific CSE blocker PGG (Fig. 4a), or silenced (Fig. 4c), apoptosis was not induced in the presence of AIK. Interestingly, when MPST was silenced, apoptosis induction was not suppressed (Fig. 4d). In order to verify a specific effect of silencers, we used also scrambled siRNA (scr) that was created against nonexisting sequence and in this group apoptosis was same as in control, untreated group, which proved the specific effect observed by silencing CBS, CSE and MPST. Effectivity of silencing of CBS was ~ 60%, CSE ~ 70% and MPST ~ 50%, as determined by gene expression. In apoptotic RCC4 cells, expression of the CBS, CSE and also MPST was increased (Fig. 5). During apoptosis, CBS and CSE immunostaining was most robust around nuclei, which was not the case of control, non-apoptotic cells. MPST signal seems to be translocated to the nuclei during apoptosis (Fig. 5). Specificity of signal was proved by performing negative control (NC), where primary antibody was omitted. We also performed Western blot analysis to verify AIK-induced increase of CBS, CSE and MPST (Fig. 5).

## Discussion

We have found that in ccRCC tumors, all three enzymes endogenously producing H_2_S are mostly downregulated compared to corresponding non-affected kidney tissue from the same patient. Since there are no data about changes of the levels of endogenous producing H2S enzymes, we performed immunohistochemistry with corresponding antibodies. We have observed that levels of the CBS and CSE is lower by the higher grade of ccRCC. Since Takano and coworkers [24] have found that reduced CBS expression promotes glioma tumorigenesis, our results are in line with their observation, since tumorigenicity increased with a grade. However, since we had just 3 tumors of a grade IV, these results had to be verified. Interestingly, in papillary carcinoma, CBS levels were not decreased as in ccRCC, but significantly increased. In angiomyolipoma, no conclusive results were obtained because of a low number of tumors.

Recently, Shackelford et al. [19] have found that CBS protein levels were increased in clear cell renal cell carcinomas (ccRCCs) in comparison to benign renal cortex and suggested that CBS and H_2_S likely play a role in malignant and benign neoplastic renal disease. For their experiments, they used two tissue microarrays, containing 29 benign renal cortical tissue samples, 57 renal urothelial carcinomas, 6 renal oncocytomas and 107 RCCs. Thus, in their experiments, control and tumor samples are not pair matched. In our study, expression of CBS, CSE and MPST in tumors was compared to the healthy – non tumorous – tissue from the same patient. This approach would eliminate potential genotypical differences, although it did not give us an idea about the expression of these enzymes in healthy part of kidney of each patient. Just to get an idea about basal expression of these enzymes in non-affected part of kidney, we compared expressions of CBS, CSE and MPST from a healthy part of kidney from patients with ccRCC and benign tumors (angiomyolipoma and oncocytoma). Surprisingly, we got 1.5–1.7- fold increase in gene expression of all H_2_S producing enzymes in healthy part of kidney from patients with ccRCC compared to patients with non-malignant tumors. Therefore, to evaluate the expression of these enzymes in tumors, it seems to us to be important to consider results according to a proper control.

In healthy kidney, H_2_S can regulate its excretory function, possibly by the inhibition sodium transporters on renal tubular cells [15]. Inhibition of either CSE or CBS in pathological states severely worsens renal damage [25]. Thus, increased levels of CBS, CSE and MPST in healthy part of kidney in patients with ccRCC might be a part of compensatory mechanism for a function of affected kidney. However, this assumption needs to be verified by more extensive studies. All three enzymes – CBS, CSE and MPST – have been present in kidney, although the significance of MPST mediated H_2_S generating pathway is still not clear [15].

Up to now, several papers described an involvement of exogenous H_2_S in the induction of apoptosis [26–30] and suggested that H_2_S is able to induce apoptosis. In the functional importance of our observation we proposed that CBS, CSE and/or MPST expression in renal cancer might be associated with the apoptosis induction. This is supported by the results obtained in RCC4 stable cell line, where we silenced individual enzymes and afterwards induced apoptosis. Expression of CBS, CSE and MPST was increased in the presence of apoptotic inducers. Apoptosis was completely abolished in a group of cells, where CBS, CSE, but not MPST was silenced. Also, the same effect was observed when CSE was blocked by a specific CSE blocker – propargyl glycine and CBS with a blocker AOAA. These results would suggest that decrease in CBS, CSE expression would aggravate the possibility of apoptosis induction in ccRCC. In ccRCC, involvement of the MPST in the induction of apoptosis was not determined, probably because MPST functions more efficiently at high pH [11].

## Conclusions

In summary, our study demonstrated that in ccRCC tumors, all three enzymes endogenously producing H_2_S are mostly downregulated compared to corresponding non-affected kidney tissue from the same patient. Levels of CBS and CSE were lower by the higher grade of ccRCC. Elucidation of the role of enzymes endogenously producing H_2_S in oncology is still uncertain. We have clearly shown that increased expression of CBS and CSE, but not MPST occurred after apoptosis induction. It become evident that besides diversity of tumors, a grade of a tumor plays an important role in evaluation expression of the CBS, CSE and MPST.

## Abbreviations

AOAA: O-(Carboxymethyl) hydroxylamine hemihydrochloride
CBS: Cystathionine β-synthase
ccRCC: Clear cell renal cell carcinoma
CSE: Cystathionine γ-lyase
H_2_S: Hydrogen sulfide
MPST: 3-mercaptopyruvate sulfurtransferase
NC: Negative control
PGG: D,L-propargyl glycine
RCC: renal cell carcinoma
